# Global consensus for sarcopenia

**DOI:** 10.18632/aging.205919

**Published:** 2024-05-17

**Authors:** Ben Kirk, Peggy M. Cawthon, Alfonso J. Cruz-Jentoft

**Affiliations:** 1 Department of Medicine, Western Health, Melbourne Medical School, University of Melbourne, Melbourne, VIC. Australia; 2 Australian Institute for Musculoskeletal Science (AIMSS), University of Melbourne and Western Health, Melbourne, VIC, Australia; 3 California Pacific Medical Center, Research Institute, San Francisco, CA 94143, USA; 4 Department of Epidemiology and Biostatistics, University of California, San Francisco, CA 94143, USA; 5 Servicio de Geriatría, Hospital Universitario Ramón y Cajal (IRYCIS), Madrid, Spain

**Keywords:** sarcopenia, global definition, GLIS, skeletal muscle

Skeletal muscle loss and weakness (Sarcopenia) is a global societal issue. This is due to the role of skeletal muscle in health and disease. Indeed, this organ provides mechanical attributes to the body necessary for maintaining posture, balance, and gait stability. Low muscle mass or low strength/function increases the susceptibility to poor outcomes such as fragility hip fractures, disability, and low quality of life in older people [1–4]. Skeletal muscle also acts as an endocrine organ and interacts with local and distal tissues; for instance, muscle cells secrete molecules involved in bone fracture healing and the same molecules help regulate distal tissues such as the brain, heart, and kidneys [5, 6]. This may partially explain why low muscle mass is a strong predictor of disease-specific mortality (dementia, cancer, heart failure, kidney/liver disease) as well as all-cause mortality in aging [7].

Until now, there has been no universal agreement on a definition for Sarcopenia. Previous definitions were continent- and region-specific: Asia, Europe, North America, and Australia/New Zealand [8]. These definitions were certainly important in drawing attention to, and understanding of, this muscle disease. However, these definitions led to wide estimates in disease prevalence/incidence as well as heterogeneity when comparing treatments results of randomised trials [9]. The lack of a single definition likely impacted the identification of or treatment for sarcopenia in research and clinical practice (i.e., caused confusion for scientists, physicians and health care professionals on which definition, cutpoints, and muscle assessment tools to employ).

To address this, the Global Leadership Initiative in Sarcopenia (GLIS) [9] was formed to create a unified global definition for use in research and clinical settings. The GLIS was formed of representatives from all sarcopenia continental consensus groups and major musculoskeletal societies and organisations worldwide (see list of societies/organisations in acknowledgment section). During the formation of GLIS, that comprised 21 steering committee members and 107 members for the full committee, efforts were made to increase balance of sex, demographic location (under-represented countries), and ethnicity. The steering committee then set pre-specified Delphi study parameters including a two-phase study design and an acceptance threshold of >80% for statements [9]. A glossary of terms on sarcopenia was also published to assist experts in answering statements (and in the hope of standardizing sarcopenia terminology in the field) [10]. These facets helped increase study rigour and transparency in reporting of outcomes.

**Figure 1:**
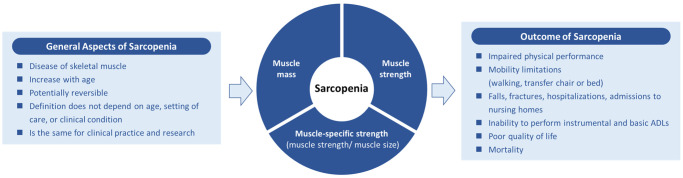
A schematic of the global conceptual definition of sarcopenia sourced from Kirk et al. [9] under the Creative Commons CC-BY-NC license.

Through a two-phase International Delphi Study, comprising of 107 academic, industry, and health professionals from 29 countries and 7 continents, a global consensus on the definition of sarcopenia was reached. This included … “6 statements on ‘general aspects of sarcopenia’ (strongest agreement: the prevalence of sarcopenia increases with age (98.3)), 3 statements on ‘components of sarcopenia’ (muscle mass (89.4%), muscle strength (93.1%) and muscle-specific strength (80.8%) should all be a part of the conceptual definition of sarcopenia) and 11 statements on ‘outcomes of sarcopenia’ (strongest agreement: sarcopenia increases the risk of impaired physical performance (97.9%)) …” [9]. The main finding of the Delphi consensus was that … “muscle mass, muscle strength and muscle-specific strength were all accepted as ‘components of sarcopenia’, whereas impaired physical performance was accepted as an ‘outcome’ rather than a ‘component’ of sarcopenia …” (see Figure 1) [9].

This conceptual definition will now serve to develop an operational definition for research and clinical settings. Of note, this unified conceptual definition of sarcopenia is expected to receive official endorsement from the World Health Organization supporting inclusion in the next International Classification of Diseases.

Overall, the development of a global conceptual definition of sarcopenia signifies a new dawn for this muscle disease. This conceptual definition will now serve as the foundation to (i), advance knowledge on the underlying mechanisms and progression of sarcopenia in biomedical research, (ii) aid epidemiological works in determining the incidence, risk factors, biomarkers, and outcomes of sarcopenia, and (iii) facilitate standardised testing of new therapeutics for sarcopenia including drug compounds, specific nutrients, and exercise regimens. In turn, reducing the societal impacts of this debilitating muscle disease and upholding the quality of life of people worldwide.
